# An Indoor Positioning-Based Mobile Payment System Using Bluetooth Low Energy Technology

**DOI:** 10.3390/s18040974

**Published:** 2018-03-25

**Authors:** Alexander Yohan, Nai-Wei Lo, Doni Winata

**Affiliations:** Department of Information Management, National Taiwan University of Science and Technology, Taipei 10607, Taiwan; d10309802@mail.ntust.edu.tw (A.Y.); m10409802@mail.ntust.edu.tw (D.W.)

**Keywords:** mutual authentication, Bluetooth beacon, Bluetooth Low Energy, indoor positioning service, mobile payment

## Abstract

The development of information technology has paved the way for faster and more convenient payment process flows and new methodology for the design and implementation of next generation payment systems. The growth of smartphone usage nowadays has fostered a new and popular mobile payment environment. Most of the current generation smartphones support Bluetooth Low Energy (BLE) technology to communicate with nearby BLE-enabled devices. It is plausible to construct an Over-the-Air BLE-based mobile payment system as one of the payment methods for people living in modern societies. In this paper, a secure indoor positioning-based mobile payment authentication protocol with BLE technology and the corresponding mobile payment system design are proposed. The proposed protocol consists of three phases: initialization phase, session key construction phase, and authentication phase. When a customer moves toward the POS counter area, the proposed mobile payment system will automatically detect the position of the customer to confirm whether the customer is ready for the checkout process. Once the system has identified the customer is standing within the payment-enabled area, the payment system will invoke authentication process between POS and the customer’s smartphone through BLE communication channel to generate a secure session key and establish an authenticated communication session to perform the payment transaction accordingly. A prototype is implemented to assess the performance of the proposed design for mobile payment system. In addition, security analysis is conducted to evaluate the security strength of the proposed protocol.

## 1. Introduction

The development of payment technology in recent years is strongly simulated by the advancement of information technology and the high throughput of network bandwidth delivered by telecommunication networks and individual Wi-Fi hotspots. According to the survey performed by European Central Bank [1], people are becoming accustomed with non-cash payment systems (either using credit or debit cards). The growth of non-cash payment systems is further driven by the integration of credit card payments with smartphone systems. Apple Pay [2], Android Pay [3], and Samsung Pay [4] are several examples of payment systems that integrate non-cash payment systems into a smartphone.

In a traditional non-cash payment system, the customer’s credit card information will be sent to the back-end payment server through an Electronic Data Capture (EDC) machine by swiping the credit card at the corresponding machine slot. For a non-cash mobile payment system, all sensitive information of a credit card is stored inside the corresponding smartphone. In general, the modern non-cash payment system utilizes the Near Field Communication (NFC) interface and embedded module of a smartphone to accomplish data exchange between the smartphone and the Point-of-Sales (POS) device [5,6]. Although NFC technology offers short service-processing time, ease of use for the customer, and simplified payment process, deployed NFC implementations on smartphone suffer from widespread non-standard payment protocols. In other words, smartphone companies only support their home-grown NFC-enabled payment systems. In consequence, merchant stores have to install multiple NFC readers to support the various proprietary payment protocols. In addition, the prices of smartphones with NFC modules are generally much higher than those of ordinary ones as the NFC module is a privileged feature for high-end smartphones and it generates extra manufacturing costs.

The development of Bluetooth technology opens up another pathway to deliver new mobile payment systems. New Bluetooth Low Energy (BLE) technology requires less energy consumption than classic Bluetooth technology [7], which allows energy-limited BLE-devices to communicate with nearby BLE devices with much less energy consumption. As most smartphones support BLE technology and are equipped with BLE communication modules, it is natural to construct a mobile payment system using BLE technology. Despite the advantages offered by BLE technology, the communication channel between the BLE-enabled smartphone and the BLE-enabled POS device has to be secured when sensitive data such as credit card information and payment amount is transmitted through the BLE-based mobile payment system.

For a BLE-based mobile payment system, it is necessary to have a robust security mechanism to defend against attacks through the payment transaction. One essential security criteria for the system is to support mutual authentication. Prevention of session hijacking is another important security requirement for mobile payment systems. A simple solution is to utilize a Bluetooth jammer [8]; however, the usage of Bluetooth jammers may cause inconvenience to customers since a Bluetooth jammer will disturb or interfere with BLE signals transmitted from other customers’ devices within its signal coverage area. To enhance security strength of user authentication, multi-factor authentication schemes have emerged in recent years. For example, face recognition technology and Bluetooth beacon technology were adopted together to construct a two-factor authentication scheme for the proposed Zero-Effort mobile payment system [9]. As facial recognition scheme generally involves with sophisticated computation and suffers with lower identification accuracy, the Zero-Effort system requires more computing resources at the backend server and consumes more time to establish an authenticated session.

In this paper, a new system design for a BLE-based mobile payment system is introduced. To secure the proposed system, a BLE-based user (customer) authentication protocol with mutual authentication features is invented. In addition, the proposed payment system utilizes Bluetooth beacon technology to accurately identify whether a customer has stepped into the payment-enabled area. Once a customer is detected in the payment-enabled area, the position information of the customer generated from the indoor positioning scheme will be adopted as a part of the user (customer) secret. The position information of customers is used to ensure the user presence during payment process. Based on the proposed authentication protocol, the user secret will be used later to authenticate the user and establish a payment session. In consequence, the proposed payment system supports mutually authenticated payment sessions for customers. In addition, an indoor positioning scheme is used to precisely identify a checking-out customer based on his position. This scheme is adopted to prevent session hijacking by malicious attackers who are not present in the payment-enabled area. Moreover, the authentication protocol dynamically constructs a session key based on the current user position information, the identity of the user’s smartphone, and the identity of the POS counter.

Based on the proposed system environment, there is a payment-enabled area in front of each POS terminal counter. Within each area, four Bluetooth beacons such as iBeacon [10] and Estimote Beacon [11] are deployed and installed along the frontage and rear of consecutive counters. When a customer steps into a payment-enabled area and is ready to pay for his purchases, he should activate the payment App installed in his smartphone. Then the App will drive the smartphone to scan for the Bluetooth beacon signals in the payment-enabled area. The smartphone (or the App) will capture beacon signals from three or four different beacon tags and use them as the input data to invoke the indoor positioning algorithm to determine the exact customer location. Once the App confirms the smartphone is inside the payment-enabled area, the App will connect with the Trusted Third Party (TTP) server and send the captured beacon signals through secure channel to retrieve a unique user secret, which is generated from the beacon signals received by TTP. The user secret will be used by the App to connect with the POS terminal associated with the identities of detected beacon tags in order to dynamically construct a session key based on a modified version of Juggling Password Authenticated Key Exchange (J-PAKE) protocol [12,13]. The contributions of this paper are as follows:

1. A new mobile payment system design using an indoor positioning-based service is proposed. In the proposed system, Bluetooth beacon technology is used to determine the current position of a checking-out customer. Based on the detected customer position, the system determines whether the customer could legitimately connect with the POS terminal corresponding to the target payment-enabled area through BLE technology, and perform check-out actions.
2. An indoor positioning-based authentication protocol using BLE technology is invented to support the proposed mobile payment system. Instead of adopting PKI-based authentication algorithms for protocol design, Shamir secret sharing and password-based key exchange algorithm are used to derive a key exchange protocol with mutual authentication feature.
3. Security analysis on the proposed protocol is conducted and a prototype is implemented to evaluate the performance of the proposed system. Based on the security analysis results, the proposed system defends against the impersonation attack, the man-in-the-middle attack, and the replay attack, and supports mutual authentication and session key security. The prototype based on BLE 4.2 takes approximately 10 s in average to accomplish one user authentication.

The remainder of this paper is organized as follows: Bluetooth technology and related literature on mobile payment systems are introduced in Section 2. The proposed design of our indoor positioning-based mobile payment system is presented in Section 3. Section 4 describes the proposed mobile payment authentication protocol. Section 5 addresses the prototype implementation and performance measurement. Security analysis for the proposed authentication protocol is presented in Section 6. Finally, the concluding remarks are presented in Section 7.

## 2. Literature Review

### 2.1. BLE and Bluetooth Beacon Technologies

Compared with classical Bluetooth, BLE technology was specifically designed to have lower energy consumption. In addition, BLE technology was specifically designed for transmission of small amounts of data, which makes it is suitable to be installed on sensors, wearable devices, and personal computers [14]. Because of these characteristics of BLE technology, it represents a viable solution for the Internet of Things [7,15,16,17].

Bluetooth beacons are a technology built on the concept of broadcasting small pieces of information in a BLE communication channel that can be detected by any supported devices. The information broadcast by a Bluetooth beacon consists of a short and simple data format, e.g., the Universal Unique Identifier (UUID) of the device, telemetry, and URL. In general, Bluetooth beacons takes a broadcaster role in the Bluetooth environment. One notable application of Bluetooth beacons is used in the indoor positioning scenario, since they can provide location information with a satisfactory level of precision [14,17,18,19,20]. In [21], Santiago et al. implemented a Smart Tourism application which utilizes Bluetooth beacons to provide information regarding specific Points Of Interest (POIs) without the availability of an Internet connection.

Generally, Bluetooth signal strength, known as Received Signal Strength Indicator (RSSI), is used to estimate the distance between the Bluetooth beacon and the receiver device. In reality, it is quite difficult to obtain good distance calculation accuracy from the reading of RSSI signal because of signal fluctuation caused by signal interferences or signal absorption, wave diffraction, multipath propagation, and other external environment factors. In order to overcome this issue, several approaches can be used to improve the distance calculation accuracy using RSSI signals, such as combining the RSSI calculation with a particle filtering method, sensor fusion and noise reduction algorithms.

Several methods could be used in indoor positioning algorithms using beacons such as trilateration, triangulation, least squares methods and fingerprinting methods [22,23]. Trilateration and triangulation computation are two common methods that have been widely used in indoor positioning algorithms. In trilateration, the absolute or relative location of a specific target, the customer’s smartphone, is obtained from distance measurements using geometric shapes, i.e., the geometry of circles is used in the case of beacons [24,25]. On the contrary, triangulation methods calculate the targeted point location by forming a triangle from known points (e.g., the beacon’s location) to the target position [26,27]. The difference between triangulation and trilateration methods lies in the distance measurement method, where triangulation only involves angle measurements rather than measuring the distance.

Least squares methods are other methods commonly used in indoor positioning algorithms [22]. These methods introduce a prediction error between the distance of an observed signal and the predicted target’s coordinate position. Iterative or repetitive measurements are used to reduce the prediction error rate in the least squares method, hence, it could give better distance measurement precision and accuracy.

Fingerprinting is another method that can be used to determine the position of target device by means of distance comparisons between the current target device positions with a pre-stored target device position reference [14,20,28]. Fingerprinting is claimed to be a more robust, accurate, and cost-effective approach. There are two phases involved in the fingerprinting method, namely the training (offline) phase and active (online) phase. In the training phase, unique attributes of the target device, e.g., RSSI, Inquiry Response Rate (IRR) or Link Quality (LQ), are recorded and labeled into grids in a two-dimensional space map; the recorded results then will be stored in the system server. In the active phase, the actual target device position is estimated and predicted by comparing the scanned device’s position with the reference position data that has been previously collected.

### 2.2. Mobile Payment System

An authentication protocol for a BLE-based mobile payment system is proposed by Yohan et al. in [8]. In Yohan et al.’s payment system, a wearable device is used to connect with wearable payment counter via a BLE interface. In order to protect the communication channel between the customer’s wearable device and the wearable payment counter, the counter is equipped with a Bluetooth jammer. The Bluetooth jammer is a directional signal jamming device built to disrupt the communication on the physical layer of Bluetooth technology [29]. Several publications [29,30] indicate that a selective (directional) jamming device can be used to construct a secure space such that communicating parties inside the space will not suffer from session hijacking and communication signal interference. However, the usage of jammers in public areas is considered illegal and requires special permission from the government in several countries [31]. Furthermore, the usage of signal jammers could bring inconveniences to other customers located outside the payment area, since the jammer could interfere with the radio signals for the other customers’ smartphones.

Smowton et al. in [9] proposed a hands-free payment system using a combination of face recognition technology, human assistance, and proximity device detection. However, the system proposed by Smowton et al. could threaten the customer’s privacy because the system needs to capture the customer’s face and send the captured data to a server for comparison process.

Zolfaghar et al. in [32] proposed a mechanism to secure Bluetooth channels using honeypots. The method proposed by Zolfagher et al. uses a fake device that appears as a valuable object and an easy target for the attackers. The honeypot system could be used as a prevention, early detection, and deterrence of malicious attacks mechanism by examining any malicious and unauthorized behaviors. In the scenario designed by Zolfaghar et al. the attacker is likely to use some brute force method to force the inquiry request until it receives an acknowledgement request from a legitimate user. In regard of this malicious request, the honeypot will be the one who responds to this kind of request and immediately initiates a connection to the attackers, thus preventing further attacks. However, this approach could not prevent attack from a trusted entity in the Point of Sale environment, such as an impersonation attack to steal a connection from a legitimate user.

According to Zhou [33], it is important to build users’ initial trust in the mobile payment system because of the risks (whether internal or external) involved in the system. Based on the experiment performed by Zhou, there are two kinds of factors that could affect the user’s trust toward a mobile payment system, namely self-perception-based factors and transference-based factors. Self-perception-based factors are defined as any element that affects the user experience regarding the mobile payment system; it also includes the expectancy that the user has toward the mobile payment system. Other factors that could affect the user’s trust toward mobile payment systems are transference-based factors. Transference-based factors are related with the legal structure that supports the mobile payment system, including the security in the payment process. Based on the research performed by Zhou, these two types of factors affect users’ trust toward mobile payment systems.

Martínez-Peláez et al. in [34] proposed a person-to-person mobile payment scheme controlled by expiration dates. In their scheme, Wireless Public Key Infrastructure (WPKI) is used to provide mutual authentication and built a secure channel to perform the authentication process and transmission of payment information. Based on their system design, the payment process is carried through a Bluetooth communication channel. In the payment system both the user and merchant need to register first at a trusted bank and request the bank to issue a certificate during the registration process. At the same time, during the transaction process, the bank would also act as Certification Authority (CA). Based on their design, the bank needs to be able to authenticate a lot of payment transaction requests and send the authentication results in a relatively short time.

Badra and Badra in [35] proposed a lightweight security protocol for NFC-based mobile payments. This approach utilizes certificate-based authentication between a POS terminal and a TTP server, and shared secret-based authentication mechanism between the POS terminal and customer’s smartphone. The customer’s secret key is stored inside the secure element of the customer’s smartphone. During the session key construction phase, either the POS terminal or the customer’s smartphone will request a session key from the TTP server. In this protocol asymmetric encryption is used during the session key exchange process between the TTP, the POS terminal and the customer’s smartphone.

## 3. The Design of Indoor Positioning-Based Mobile Payment System

### 3.1. Assumptions

Several assumptions used in the proposed mobile payment system are listed as follows:

1. Both customer and merchant have a device embedded with BLE technology. In the proposed system design, the customer uses a BLE-enabled wearable device such as smartphone and smart-watch, and the merchant device is a BLE-enabled POS terminal. Before a connection between the customer’s smartphone and the POS terminal is established, the smartphone takes the role of the observer and the POS is the advertiser in the BLE environment. Once the BLE connection is established, the smartphone serves as the master device, while the POS takes on the role of slave device.
2. Each POS terminal has its own payment area, which is equipped with three to four beacon tags. These beacon tags broadcast signals and information that are used by the customer’s smartphone to detect whether the customer is located in the payment-enabled area of a specific POS terminal associated with the corresponding beacon tags.
3. Each Bluetooth beacon tag in the payment area stores a unique identifier, POS terminal identifier, and a partial POS secret. A Shamir secret sharing scheme is used in advance to divide each POS terminal secret into four parts. These four partial secrets are then stored in four corresponding beacon tags of the specific POS terminal. The POS unique identification, the POS secret, and the four corresponding beacon tag identifiers are also stored on the TTP server. The TTP server is only accessible by authorized applications such as the mobile payment system and the corresponding registered POS terminals.
4. Each Bluetooth beacon tag continuously broadcasts its unique identifier and the partial POS secret stored inside each beacon tag.
5. During the checkout process, only one customer is allowed to be inside the payment-enabled area at a time. In addition, the customer should not leave the payment-enabled area until the transaction is finished.
6. In the proposed scenario, the customer needs to register his credit card information first and obtain a unique payment token and a secret symmetric key that are stored in the secure element of his wearable device.
7. The communication channel between the customer’s wearable device and the POS terminal is not secure. The communication channel between the TTP server and the communicating party, such as the customer’s wearable device and the POS terminal, is secure. The communication channel between the POS terminal and the backend payment-processing server is secure.

### 3.2. System Architecture

**Figure 1:**
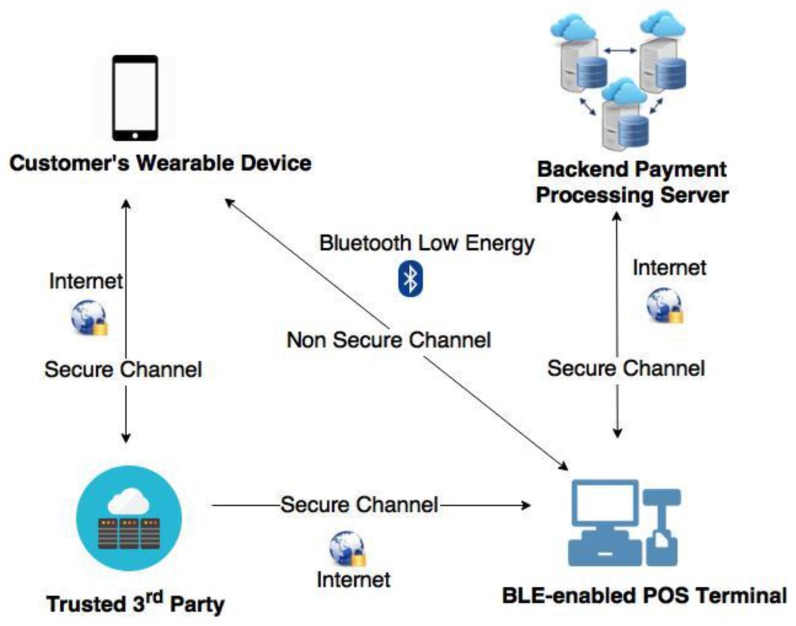
Proposed system architecture for indoor positioning-based mobile payment system.

The system architecture for the proposed protocol is depicted in Figure 1. The proposed system architecture comprises of four components: TTP server, the customer’s wearable device (such as a smartphone), BLE-enabled POS terminal, and backend payment-processing server.

In the proposed system, the TTP server is used during the initialization phase of the payment process and holds the key role to generate and distribute unique secret data to the POS terminal and the customer’s smartphone. The TTP server stores the POS terminal ID and the corresponding IDs of beacon tags. The TTP server constructs a unique secret based on the customer’s position data, the timestamp from the customer’s wearable device and the detected IDs of corresponding beacon tags sent by the user’s device. This unique secret is used by the user’s smartphone to establish a connection with the POS terminal.

The second component in the proposed system architecture is the customer’s wearable device. A BLE-based payment APP should be installed in the customer’s device in advance. Once the user (customer) enters the payment-enabled area and invokes the APP; the App automatically executes its indoor positioning algorithm module to determine the user’s position against the payment-enabled area. If the App determines that the user is located inside the payment-enabled area, then the App will initialize a BLE connection to the BLE-enabled POS terminal to proceed the payment process.

BLE-enabled POS terminal is the third component in the proposed system architecture. Each POS device that supports the proposed BLE-based mobile payment system has its unique payment area called payment-enabled area, which is equipped with three to four Bluetooth beacon tags. Each beacon tag constantly broadcasts Bluetooth signals containing the beacon tags’ ID and the partial secret of the associated POS terminal.

A backend payment processing server is the last component in the proposed system architecture. The backend payment server manages and processes all the payment transactions made by the customer wearable devices and the POS terminals. Since the proposed BLE-based mobile payment system utilizes tokenization technology to secure the payment transactions, therefore, we assume tokenization module is implemented in the backend payment server. As the payment transaction mechanism using tokenization technology is already mature, the proposed mobile payment system could directly adopt previously published payment transaction mechanisms [36,37]. Notice that for the sake of simplicity, the term wearable device and smartphone are used interchangeably for the rest of this paper.

### 3.3. System Flow Design

**Figure 2:**
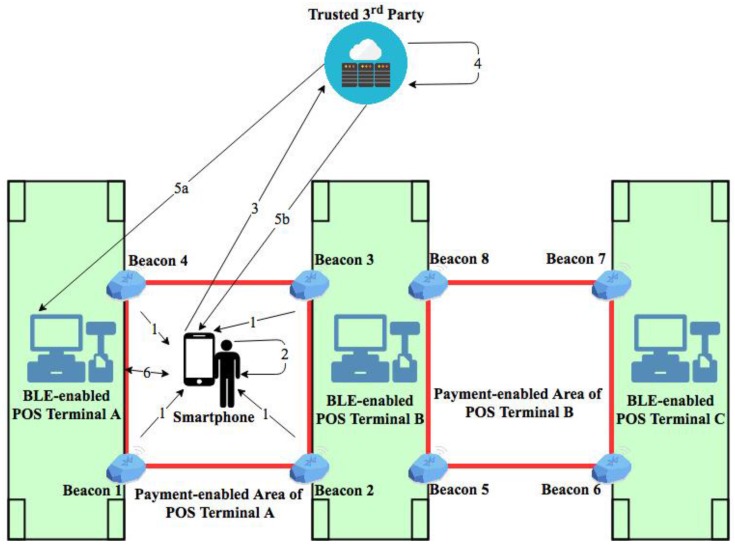
Process flow of the proposed BLE-based mobile payment system is categorized into 6 steps: (1) advertisement of beacon tag information; (2) user’s position detection; (3) transmission of user position to TTP server; (4) construction of user secret; (5a) distribution of user secret to the corresponding POS terminal; (5b) distribution of user secret to the user’s smartphone, and (6) mutual authentication between the user’s smartphone and the POS terminal to establish a secure payment session for the payment transaction.

The system flow of the proposed BLE-based mobile payment system depicted in Figure 2 is explained as follows:

1. When a user (customer) is going to checkout his purchase, he invokes the BLE-based payment App installed in his device. Once a user is positioned inside a payment-enabled area of the corresponding payment counter, the App will automatically receive the advertising signals broadcast by the corresponding Bluetooth beacon tags within the payment-enabled area.
2. The payment App in the user’s smartphone executes its indoor positioning module to determine the relative smartphone position against the fixed positions of beacon tags. The partial POS terminal secrets stored in each beacon tag, the calculated RSSI value of each beacon tag and the unique ID of each beacon tag are collected and utilized in the indoor positioning module. Notice that in the initialization phase of the payment system, a partial secret of each POS terminal is pre-installed into each beacon tag associated with its corresponding POS terminal. The detail of the partial secret stored in each beacon tag is described in Section 4.1.
3. Once the user’s smartphone is detected and determined to be inside the legal payment-enabled area, the smartphone (or the App) sends a user secret generation request to the TTP server. The user secret generation request consists of the user ID, the timestamp from the user smartphone and the collected partial POS terminal secrets from four beacon tags.
4. After the TTP server has received the user smartphone request, the TTP server identifies the user’s position by matching the received partial POS terminal secrets with the registered POS terminal secret data. Then, the TTP server dynamically generates a unique user secret based on the received user information, the retrieved POS terminal secret, and the timestamp from the user smartphone. The generated user secret will be used to establish a mutually authenticated session between the user’s smartphone and the corresponding POS terminal.
5. After the TTP server dynamically generated the unique user secret, then the TTP server will distribute the user secret to the user’s smartphone and the corresponding POS terminal as shown in (5a) and (5b) of Figure 2.
6. Both the user’s smartphone (the payment App) and the POS terminal individually construct a session key based on the received user secret. The constructed session key will be used to establish a secure payment session between the user’s smartphone and the POS terminal to perform the payment transaction. The processes of user secret generation, user secret distribution, and the session key construction are performed implicitly while the customer is taking out his purchased items for the checkout process. Once the customer has decided to pay his purchase using the mobile payment system, the customer needs to authorize the payment transaction from the payment App. After the user authorize the payment transaction, a mutual authentication process is performed between the user’s smartphone and the POS terminal to activate the payment transaction. Notice that the user’s smartphone (the payment App) monitors the signals of four beacon tags continuously and implicitly to determine the user position against the payment-enabled area. If the customer steps outside the payment-enabled area before he authorizes his payment transaction in the payment App, then the payment App will abort the current payment session.

## 4. Proposed Mobile Payment Authentication Protocol

In this section, the proposed indoor positioning-based authentication protocol is addressed. There are three phases in the proposed authentication protocol: initialization phase, session key construction phase, and authentication phase. Notations used in this paper are listed in Table 1.

### 4.1. Initialization Phase

Assume the customer activates the payment App before he walks to one of the BLE-enabled POS terminals. Once the customer enters the payment area of a BLE-based POS terminal, the payment App starts to get the session secret $K_{a}$. First, the customer’s wearable device observes the surrounding environment to obtain a set of information $A_{i}^{p}$ broadcast by the four beacon tags associated with the POS terminal. Then, the collected information is sent from the customer’s wearable device to the TTP server. 

**Figure 3:**
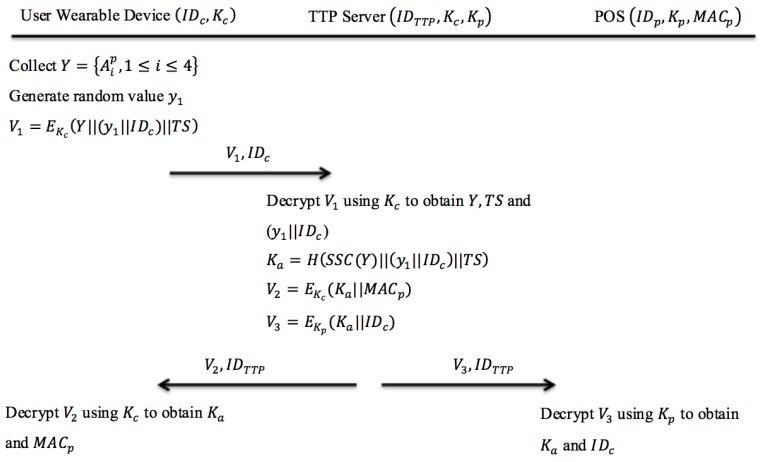
The protocol design of initialization phase.

In order to construct the secret $K_{a}$, the TTP server utilizes Shamir secret sharing method [38] to identify which POS terminal that the customer’s wearable device could connect to. In this phase, both the user’s (customer) wearable device and the POS terminal will receive a secret $K_{a}$ from the TTP server. Figure 3 shows the initialization protocol in the proposed system.

The initialization phase is described as follows:

Step 1: User Wearable Device → TTP Server: $V_{1},ID_{c}$

After the customer’s wearable device collects a set of information broadcast from beacon tags $Y=\left\{A_{i}^{p},\;1\leq i\leq 4\right\}$ associated with the POS terminal p, the wearable device generates a random value $y_{1}$. The wearable device encrypts this message $\left(Y\left|\left|\left(y_{1}||ID_{c}\right)\right|\right|TS\right)$ using the symmetric key $K_{c}$. Then the wearable device will send the encrypted message $V_{1}$ to the TTP server along with the device’s identifier $ID_{c}$.

Step 2: Internal processing at TTP server

After receiving the encrypted message $V_{1}$ from the customer’s wearable device, the TTP server will decrypt it using $K_{c}$ to obtain Y, TS and $\left(y_{1}||ID_{c}\right)$. Based on the received set of beacon information Y, the TTP server uses Shamir secret sharing construction function SSC() to obtain the corresponding POS terminal secret. By using the computed POS terminal secret, the TTP server searches its database to find the corresponding POS terminal identity $MAC_{p}$. Afterwards, the TTP server constructs a user secret $K_{a}=H\left(SSC\left(Y\right)\left|\left|\left(y_{1}||ID_{c}\right)\right|\right|TS\right)$ for the current session. After computing $K_{a}$, the TTP server will encrypt two messages $V_{2}$ and $V_{3}$. Then, the TTP server sends both $V_{2}$ and $V_{3}$ along with the identity of the TTP server $ID_{TTP}$ to the user’s wearable device and the POS terminal as shown in Step 2a and Step 2b.

Step 2a: TTP Server → User Wearable Device: $V_{2},ID_{TTP}$

The TTP server encrypts this message $\left(K_{a}||MAC_{p}\right)$ using the symmetric key of the customer’s wearable device $K_{c}$. The encrypted message $V_{2}$ is sent to the customer’s wearable device along with the identity of TTP server $ID_{TTP}$. After the customer’s wearable device receives $V_{2}$ from the TTP server, it will be decrypted using $K_{c}$ to obtain the values of $K_{a}$ and $MAC_{p}$, in which both values will be temporarily stored in the user’s wearable device.

Step 2b: TTP Server → POS: $V_{3},ID_{TTP}$

The TTP server encrypts this message $\left(K_{a}||ID_{c}\right)$ using the corresponding POS’ symmetric key $K_{p}$. The encrypted message $V_{3}$ is sent to the POS terminal along with the identity of TTP server $ID_{TTP}$. After the POS terminal receives $V_{3}$, it will be decrypted using POS terminal’s symmetric key $K_{p}$ to obtain the values of $K_{a}$ and $ID_{c}$, in which both values will be temporarily stored in the POS terminal.

### 4.2. Session Key Construction Phase

At the end of the initialization phase, both the customer’s wearable device and the POS terminal will automatically enter the session key construction phase. In the beginning of session key construction phase, the customer’s wearable device initiates a BLE connection to the POS terminal using the user secret $K_{a}$ and the MAC address of POS terminal $MAC_{p}$. After the POS terminal receives the connection request from the customer’s smartphone, it will verify the customer’s wearable device $ID_{c}$ and the secret data $K_{a}$ sent by the customer’s wearable device. Then both the POS terminal and the customer’s smartphone use Zero Knowledge Proof functions to individually construct partial secrets which will be utilized to construct a full session key later. Then, these partial secrets will be exchanged between the customer’s wearable device and the POS terminal. With the received partial secret information, both sides could successfully construct a full session key for the current payment transaction.

To practically execute this session key construction protocol, both the customer’s smartphone and the POS terminal should have pre-installed the common parameters (G,g), where G denotes a subgroup of cyclic multiplicative group $Z_{p}^{*}$ and g is the generator of G. In order to construct a unique session key, a modified J-PAKE protocol [12,13] is derived and utilized in the session key construction phase. The modified J-PAKE protocol uses a unique and dynamic secret data instead of static password used in original J-PAKE protocol. 

**Figure 4:**
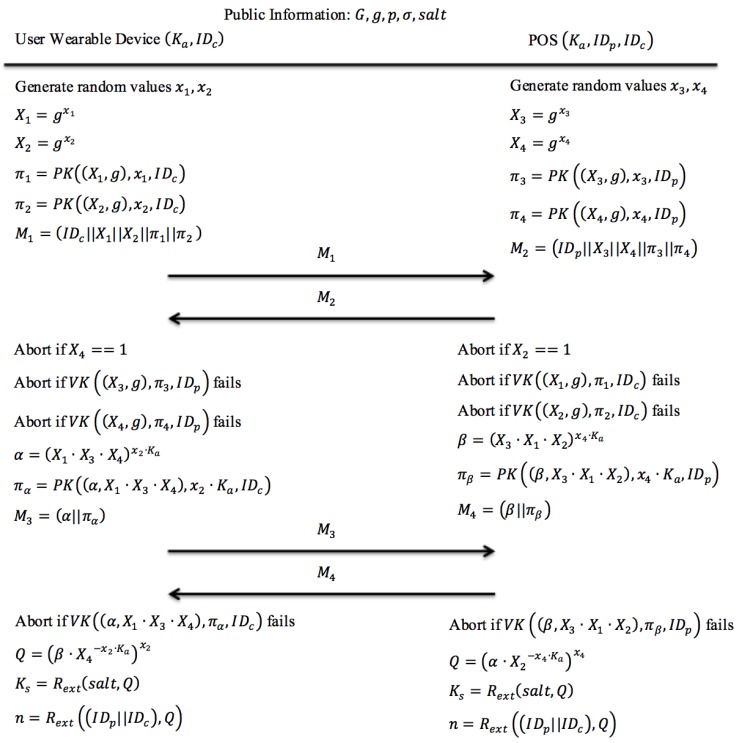
The proposed protocol design for the session key construction phase.

Figure 4 illustrates the modified J-PAKE protocol used in the proposed session key construction phase:

Step 1: Each party generates two random numbers based on the constructor g and two result values by applying the Zero Knowledge Proof function. Then both parties exchange the newly generated Zero Knowledge Proof.

The user’s smartphone generates two random values ($x_{1}$ and $x_{2}$) and computes $X_{1}=g^{x_{1}}$, $X_{2}=g^{x_{2}}$, and 2 zero knowledge proofs $\pi_{1}=PK\left(\left(X_{1},g\right),x_{1},ID_{c}\right)$ and $\pi_{2}=PK\left(\left(X_{2},g\right),x_{2},ID_{c}\right)$. At the same time, the POS terminal also generates two random values ($x_{3}$ and $x_{4}$) and computes $X_{3}=g^{x_{3}}$, $X_{4}=g^{x_{4}}$, and 2 zero knowledge proofs $\pi_{3}=PK\left(\left(X_{3},g\right),x_{3},ID_{p}\right)$, and $\pi_{4}=PK\left(\left(X_{4},g\right),x_{4},ID_{p}\right)$. Next, the user’s smartphone sends a message $M_{1}$ to the POS terminal. Likewise, the POS terminal sends a message $M_{2}$ to the user’s smartphone.

Step 2: Each party verifies the received Zero Knowledge Proof and constructs a new Zero Knowledge Proof based on the previously exchanged proof. Then both parties exchange the newly generated Zero Knowledge Proof again.

Both the user’s smartphone and the POS terminal verify the received zero knowledge proofs in the messages $M_{1}$ and $M_{2}$, respectively. Once the received zero knowledge proofs have been verified, the user’s smartphone computes the value $\alpha ={\left(X_{1}\cdot X_{3}\cdot X_{4}\right)}^{x_{2}\cdot K_{a}}$ and its zero knowledge proof $\pi_{\alpha }=PK\left(\left(\alpha ,X_{1}\cdot X_{3}\cdot X_{4}\right),x_{2}\cdot K_{a},ID_{c}\right)$. Meanwhile, the POS terminal computes the value $\beta ={\left(X_{3}\cdot X_{1}\cdot X_{2}\right)}^{x_{4}\cdot K_{a}}$ and its zero knowledge proof $\pi_{\beta }=PK\left(\left(\beta ,X_{3}\cdot X_{1}\cdot X_{2}\right),x_{4}\cdot K_{a},ID_{p}\right)$. Afterward, the user’s smartphone sends a message $M_{3}$ to the communicating POS terminal. Similarly, the POS terminal sends a message $M_{4}$ to the user’s smartphone.

Step 3: Each party verifies the received Zero Knowledge Proof and constructs a new session key by applying the random string extension function with the public parameter salt, and the calculated value Q. The calculated value Q is constructed from user secret $K_{a}$ and the verified random values $\left(X_{1},X_{2},X_{3},X_{4}\right)$ from both parties.

Both the user’s smartphone and the POS terminal verify the received values of zero knowledge proofs in the messages $M_{3}$ and $M_{4}$, respectively. After the received zero knowledge proof values have been verified, the user’s smartphone calculates a secret value $Q={\left(\beta \cdot X_{4}{}^{-x_{2}\cdot K_{a}}\right)}^{x_{2}}$ and the POS terminal calculates a secret value $Q={\left(\alpha \cdot X_{2}{}^{-x_{4}\cdot K_{a}}\right)}^{x_{4}}$. Both the user’s smartphone and POS terminal computed value Q should be equivalent based on the characteristic of Zero Knowledge Proof. The secret value Q is used to compute session key $K_{s}=R_{ext}\left(salt,Q\right)$ and nonce $n=R_{ext}\left(\left(ID_{p}||ID_{c}\right),Q\right)$. Both parties will temporarily store the generated session key $K_{s}$ and the nonce n for later usage during the authentication phase.

### 4.3. Authentication Phase

**Figure 5:**
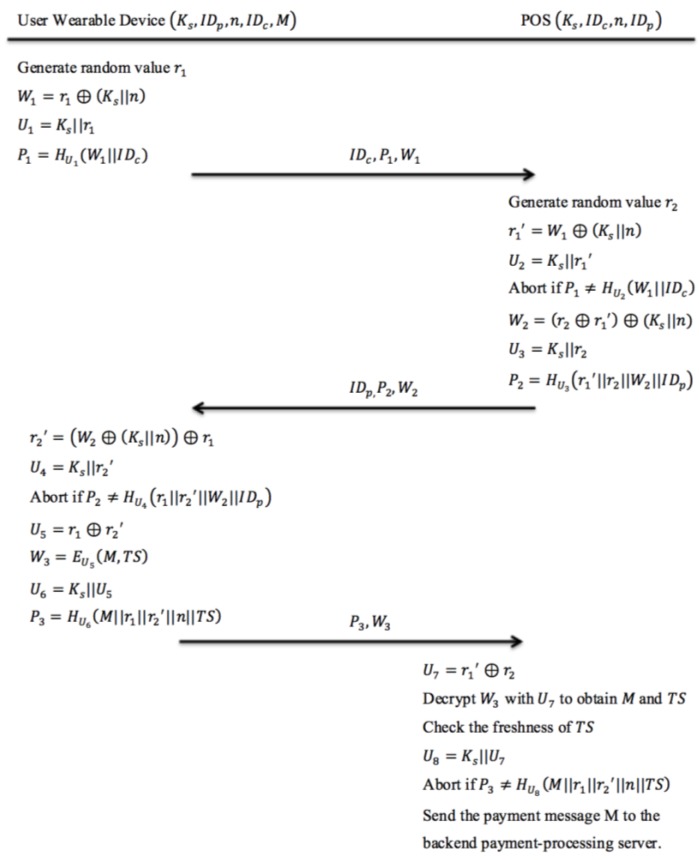
The proposed authentication protocol for BLE-based mobile payment system.

Once the customer confirms to pay his purchase through the payment App interface, the payment App initiates the authentication phase immediately before transmitting the user’s payment information to the backend payment-processing server. The proposed authentication protocol is shown in Figure 5.

Step 1: User Wearable Device → POS: $ID_{c},P_{1},W_{1}$

First, the customer’s smartphone generates a random value $r_{1}$ and uses $r_{1}$ to compute $W_{1}=r_{1}⊕\left(K_{s}||n\right)$ and $U_{1}=K_{s}||r_{1}$. The newly computed $U_{1}$ is used as the hash key for the following hash computation $P_{1}=H_{U_{1}}\left(W_{1}||ID_{c}\right)$. Then the smartphone will send both $P_{1}$ and $W_{1}$ to the POS along with the smartphone’s identity $ID_{c}$.

Step 2: POS → User Wearable Device: $ID_{p},P_{2},W_{2}$

After the POS receives $P_{1}$ and $W_{1}$, it generates a random value $r_{2}$. Afterwards, the POS will calculate $r_{1}{}^{'}=W_{1}⊕\left(K_{s}||n\right)$ and use it to compute the hash key $U_{2}=K_{s}||r_{1}{}^{'}$. After computing $U_{2}$, the POS compares the received value $P_{1}$ with the calculated value $H_{U_{2}}\left(W_{1}||ID_{c}\right)$ to verify the freshness and the correctness of the session key $K_{s}$. If the verification test passes, then the POS will calculate the value $W_{2}=\left(r_{2}⊕r_{1}{}^{'}\right)⊕\left(K_{s}||n\right)$. Otherwise, the authentication process will be aborted. After computing $W_{2}$, the POS calculates another hash key $U_{3}=K_{s}||r_{2}$ and uses it to generate a hashed value $P_{2}=H_{U_{3}}\left(r_{1}{}^{'}\left|\left|r_{2}\right|\right|W_{2}||ID_{p}\right)$. Afterwards, the values $P_{2}$ and $W_{2}$ are sent to the customer’s smartphone along with the POS terminal identity $ID_{p}$.

Step 3: User Wearable Device → POS: $P_{3},W_{3}$

When the smartphone receives $P_{2}$ and $W_{2}$, it will compute $r_{2}{}^{'}=\left(W_{2}⊕\left(K_{s}||n\right)\right)⊕r_{1}$ and $U_{4}=K_{s}||r_{2}{}^{'}$. After computing $r_{2}{}^{'}$ and $U_{4}$, the customer’s smartphone will compare the received value $P_{2}$ with the hashed value $H_{U_{4}}\left(r_{1}||r_{2}{}^{'}\left|\left|W_{w}\right|\right|ID_{p}\right)$. If both values are equivalent, then the customer’s smartphone computes a new hash key $U_{5}=r_{1}⊕r_{2}{}^{'}$. Otherwise the authentication process is aborted. After computing a new hash key $U_{5}$, the customer’s smartphone will calculate the encrypted message $W_{3}=E_{U_{5}}\left(M,TS\right)$ and calculate a new hash key $U_{6}=K_{s}||U_{5}$. Notice that M is a message containing information of the current payment transaction and TS is the current timestamp generated by the user’s smartphone. The newly computed hash key $U_{6}$ is used to compute $P_{3}=H_{U_{6}}\left(M\left|\left|r_{1}\right|\right|r_{2}{}^{'}\left|\left|n\right|\right|TS\right)$. Next, both the hashed value $P_{3}$ and the encrypted message $W_{3}$ are sent to the POS.

Step 4: Internal processing at POS

In the final step of the authentication process, the POS creates a decryption key $U_{7}=r_{1}{}^{'}⊕r_{2}$ after receiving both $P_{3}$ and $W_{3}$ from the customer’s smartphone. Afterwards, the POS will decrypt the received $W_{3}$ using $U_{7}$ to obtain M and TS. After obtaining TS, the POS will check whether the value of TS is within an acceptable time duration based on its own system clock. Otherwise, the process will be aborted. Then the POS will compute a new hash key $U_{8}=K_{s}||U_{7}$ and use it to compare the received hash value $P_{3}$ with the computed hash value $H_{U_{8}}\left(M\left|\left|r_{1}\right|\right|r_{2}{}^{'}\left|\left|n\right|\right|TS\right)$. If both values are equivalent, then the POS will send the message M that contains customer’s payment information to the backend payment-processing server.

## 5. Prototype Implementation and Experiments

A prototype of the proposed mobile payment system was implemented. The POS terminal App prototype was deployed on a Samsung Galaxy S5 phone, while the mobile payment App prototype was installed on a Samsung Galaxy S6. For the Bluetooth beacon tag, iBeacon-based tags are used in the prototype. Other Bluetooth beacon protocols such as Eddystone, AltBeacon, and FatBeacon could be utilized to build similar mobile payment prototype systems. All devices used in the experiments and some of their features are listed in Table 2.

### 5.1. Prototype Implementation

**Figure 6:**
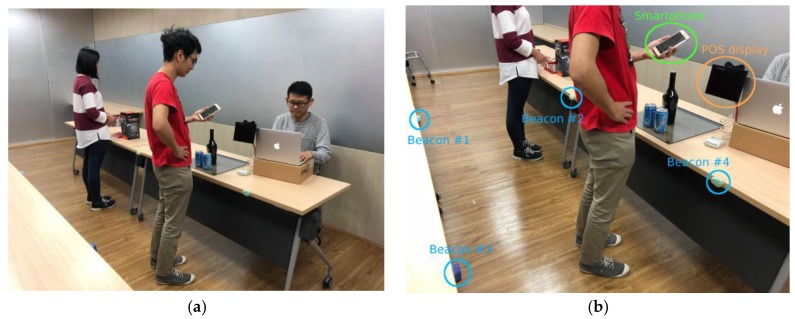
The practical implementation of the proposed mobile payment system: (**a**) four Bluetooth beacon tags are placed at the same height horizontal surface; and (**b**) a closer look on the arrangement of each device.

In our prototype, four Estimote proximity beacon tags are placed separately in each corner of a square surface with the distance between each neighboring beacon tag set to 75 cm by 75 cm. Figure 6 shows a photograph of practical implementation of the proposed mobile payment system. As shown in Figure 6a, each beacon tag is placed on a horizontal surface at the same height. In addition, each beacon tag is configured as follows:

- Advertising interval: 300 ms
- Transmitting power (Tx): −20 dBm
- Maximum signal range: up to 3.5 m (11.48 ft.).

**Figure 7:**
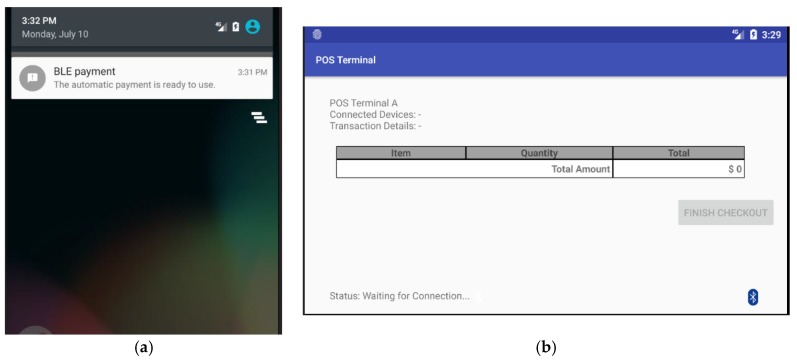
Screenshots of implemented prototype: (**a**) the notification message sent by the payment App executed as an agent process in the customer’s smartphone; and (**b**) the default screen of the POS terminal waiting for incoming BLE connection from a payment App.

Notice that the maximum signal range of a Bluetooth beacon tag is up to 3.5 m, however, the range could be set to 1 m (3.28 ft.) by setting the signal transmitting power of the beacon tag. Google’s Firebase platform is used to imitate the TTP server. In addition, the prototype of payment App includes a JSON file that contains API key to access the database in Firebase. Figure 7a shows a display of notification message sent by the payment App, which is executed as an agent process in the customer’s smartphone. Figure 7b shows the default display of POS terminal while it is waiting for incoming BLE connection from the payment App. 

**Figure 8:**
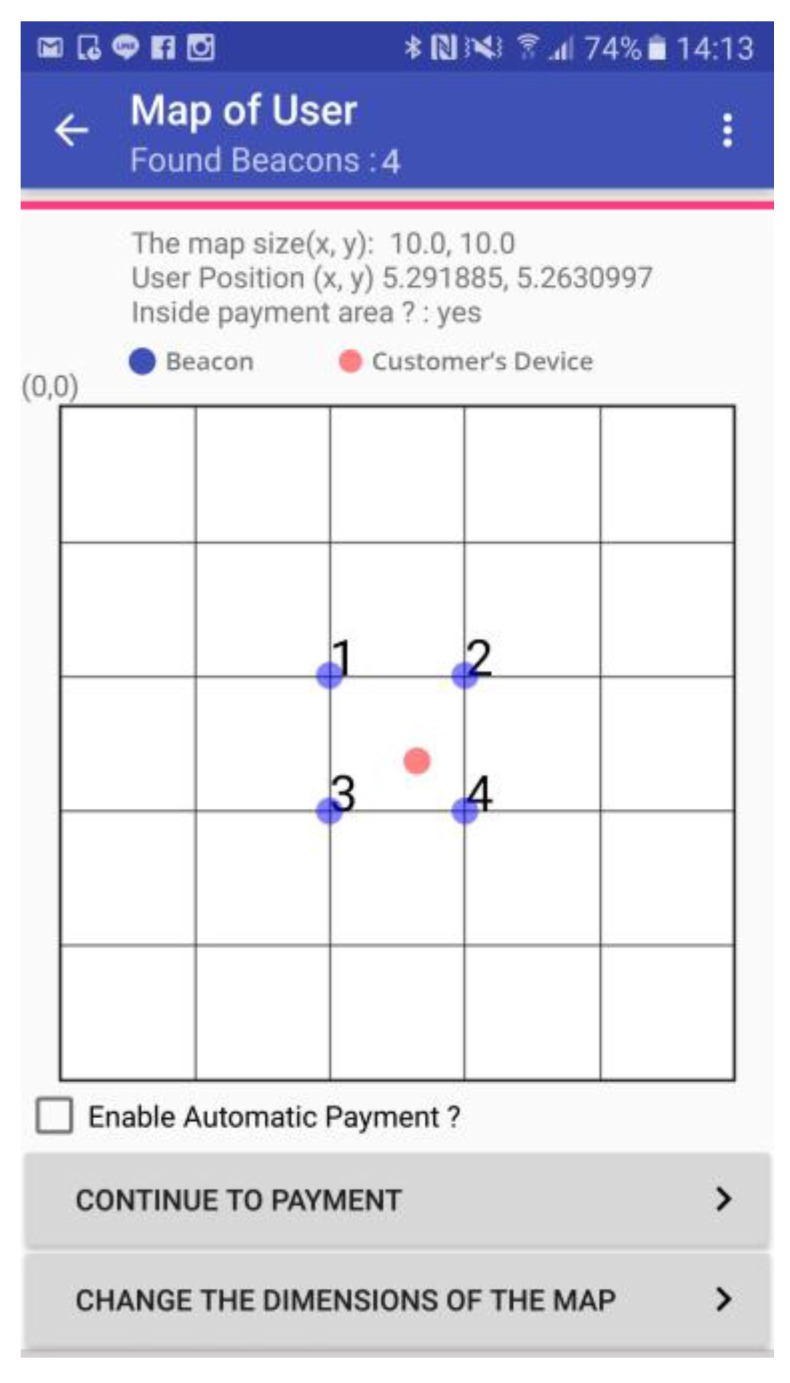
The position-monitoring App shows the current position of the corresponding smartphone in the map of a target payment-enabled area.

During our experiments, the POS terminal (Samsung Galaxy S5) is placed beside the payment-enabled area composed by the four beacon tags. In addition, a position-monitoring App is developed to detect whether the position of the customer’s smartphone is located in the payment-enabled area. The position-monitoring App shown in Figure 8 displays the position of the user’s device against the positions of four beacon tags. The blue dots shown in the map represent the beacon tags, while the red dot indicates the position of the customer’s smartphone.

Two indoor positioning algorithms are implemented in the payment App: least squares algorithm and trilateration algorithm. Once the customer enters the payment-enabled area, the user’s smartphone (or the payment App) will automatically capture the broadcast signals from the four beacons tags. This event invokes the proposed mobile payment system. Afterwards, the prototype payment App executes the indoor positioning algorithm to determine the user device position. After the payment App obtains the user secret constructed by the TTP server, the payment App will establish a BLE connection to the POS terminal which also receives the same user secret from the TTP server.

**Figure 9:**
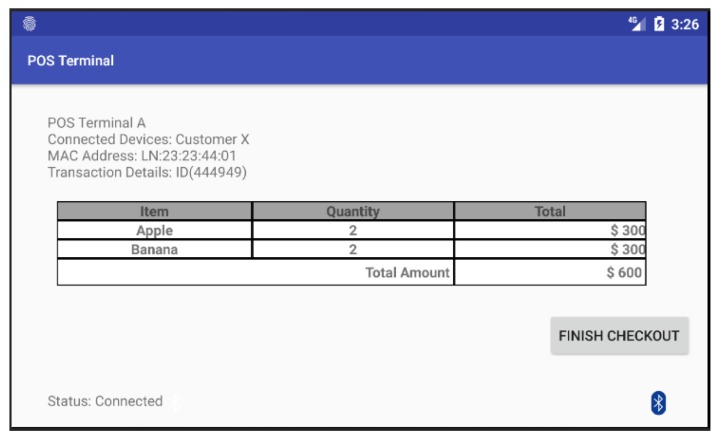
The screen of POS terminal in which the POS terminal has already connected with the customer’s smartphone and ready to execute the payment process.

**Figure 10:**
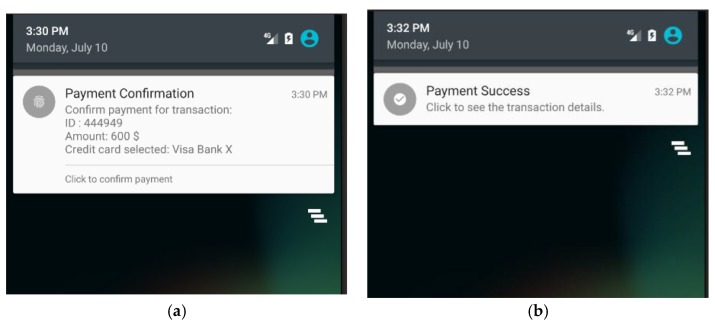
The screenshots of the payment App: (**a**) the payment App receives a payment-confirmation message sent from the POS terminal; and (**b**) the payment App has successfully processed the payment transaction.

**Figure 11:**
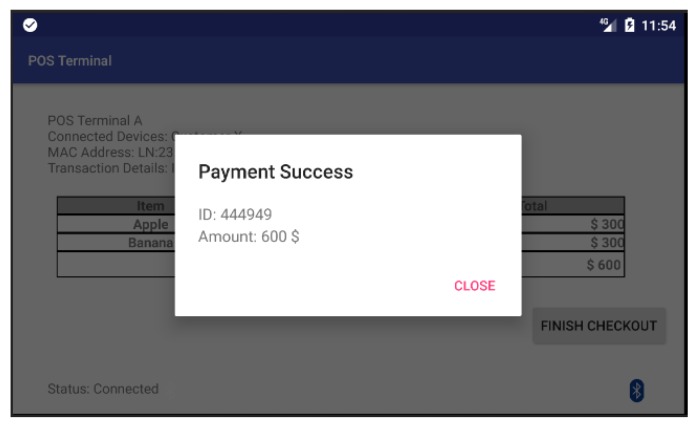
The POS terminal screen displays a notification of successful payment transaction in our prototype system.

After the customer’s smartphone establishes a BLE connection to the POS terminal, both parties start to construct the session key individually. Figure 9 shows the screen of POS terminal in which the POS terminal has already connected with the customer’s smartphone and ready to execute the payment process. When the customer clicks on the “Finish checkout” button in the touch screen of the POS terminal, the POS terminal will send a payment-confirmation message to the payment App as shown in Figure 10a. Once the customer receives the payment-confirmation message and clicks the payment confirmation button in the payment App, the authentication phase of the proposed protocol is executed to mutually authenticate both the customer’s smartphone and the corresponding POS terminal. Then, the payment transaction process is activated by transmitting session-key-encrypted payment transaction messages from the payment App to the POS terminal. Afterwards, both the payment App and the screen of the POS terminal will display a notification message regarding the current payment transaction as shown in Figure 10b and Figure 11.

### 5.2. Experiment Results

Several experiments are conducted on the prototype to assess the accuracy of the indoor positioning functionality and the protocol’s performance in terms of time consumption. The location-positioning accuracy of both least square algorithm and trilateration algorithm implemented in the payment App are evaluated in terms of the distance difference between the real position and the detected position of the customer’s smartphone. The accuracy rate for a target indoor positioning algorithm is defined by the following formula $A_{c}=\sum_{i=1}^{n}d_{i}^{c}/n$. Variable c denotes the observation region, which has the size of payment-enabled area (i.e., 75 cm by 75 cm in our experimental case). The total number of experiments is symbolized with variable n. Variable $d_{i}^{c}$ represents the prediction result of the i-th experiment based on the observation region c, in which the value of $d_{i}^{c}$ is set to 1 if the real position of the user’s smartphone is correctly predicted by the selected indoor-positioning algorithm in terms of whether the user’s smartphone is located within the observation region c or not. The value of $d_{i}^{c}$ is set to 0 if the real position of the user’s smartphone is wrongly predicted by the selected indoor-positioning algorithm. The accuracy rate $A_{c}$ is defined as the number of times for correctly predicting the position of the user’s smartphone over the total number of experiment times. To evaluate the performance of the prototype, the time consumption to establish a session is measured and a detailed time distribution for each phase within a session is analyzed.

As a user may accidentally trigger the payment App installed on his/her smartphone while he/she is still shopping around in the store, it is necessary to adopt indoor-positioning sensor technology to precisely identify the user’s location in order to confirm user’s intention to check-out. In addition, by adopting indoor-positioning sensor technology the proposed mobile payment system does not need to use a Bluetooth jammer to secure the user’s Bluetooth connection with the POS counter. In the proposed mobile payment system, once the user steps outside the payment-enabled area, then the payment App will directly terminate the payment process and abort the current session. In order to provide secure payment functionalities, the proposed mobile payment system heavily relies on the accuracy of indoor positioning sensor technology.

In order to assess the accuracy of indoor positioning function, thirty seven times of position-detection experiments for customer’s smartphone are conducted. In each experiment, the payment App will collect around thirty points of predicted position for the user smartphone based on the selected indoor positioning algorithm. After that, the payment App calculates the average value from these collected points and determines whether the user smartphone is currently located inside the payment area or not.

**Figure 12:**
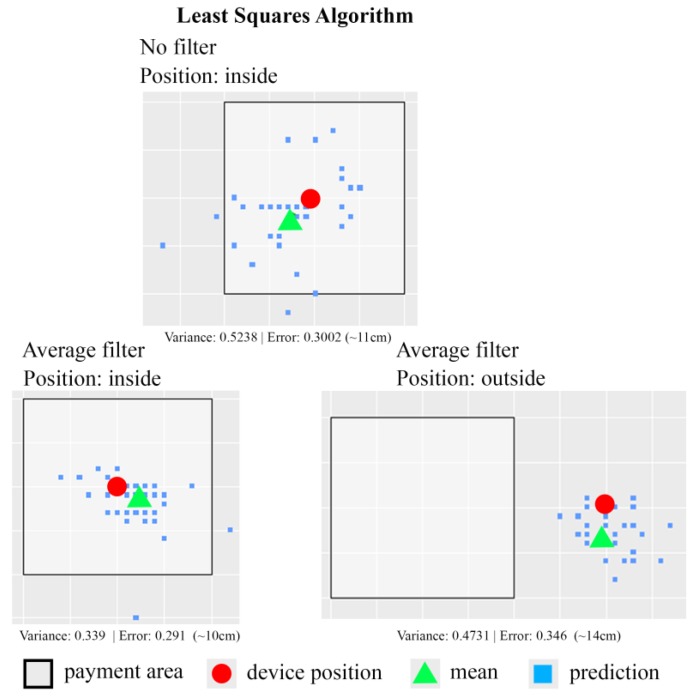
A sample of one experiment result for estimation of customer smartphone position using least squares algorithm.

The experimental results using a least squares algorithm are shown in Figure 12. The size of the square area shown in Figure 12 is 75 cm by 75 cm. In those experiments, two test strategies are used to detect the position of the customer’s smartphone: one without applying any filter and the other using the average filter. Note that the average filter indicates the averaged position value of all the computed positions of the customer’s smartphone is calculated and returns as the final estimated position. Based on the experiment results, it is clear that the indoor-positioning function implemented with the least squares algorithm plus the average filter performs better than the one without applying any filter. The error of distance measurement between the real device position and the detected device position is around 10 cm to 14 cm. Table 3 shows the experiment data from 148 experiments using least squares algorithm. Based on the experiment data, the least squares algorithm-based positioning function associated with the average filter has 97.29% of accuracy rate. The least squares algorithm-based positioning function without any filter can only achieve 93.24% of accuracy rate.

**Figure 13:**
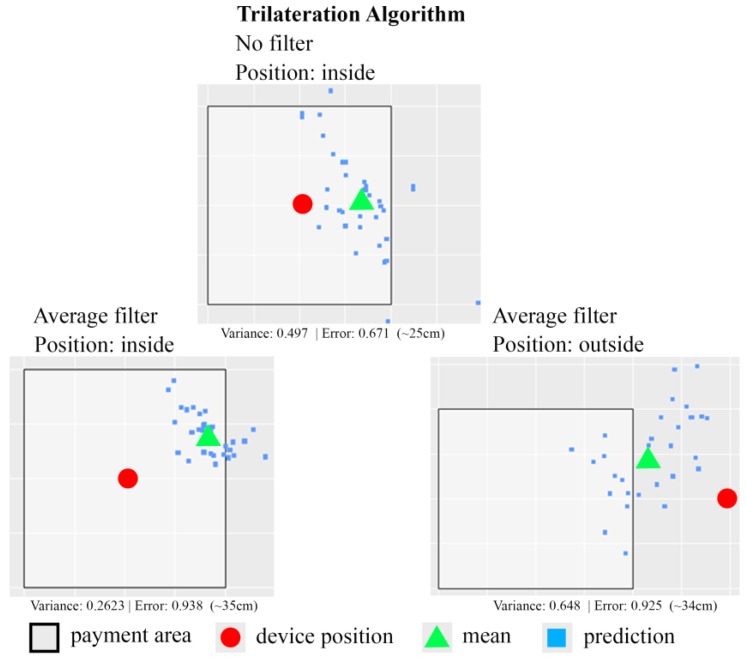
A sample of one experiment result for estimation of customer smartphone position using trilateration algorithm.

The experimental results using a trilateration algorithm are shown in Figure 13. The size of square area shown in Figure 13 is 75 cm by 75 cm. Similarly, two test strategies, one without applying any filter and the other using the average filter, are applied. In general, the positioning function using the trilateration algorithm does not produce better results than the one using the least squares algorithm. The error of distance measurement between the real device position and the detected device position is around 25–35 cm. Table 4 shows the experiment data from 148 experiments in each case using trilateration algorithm. Based on the experiment data, the trilateration algorithm-based positioning function without any filter has 78.37% of accuracy rate. The trilateration algorithm-based positioning function associated with average filter can only achieve 72.97% of accuracy rate.

**Figure 14:**
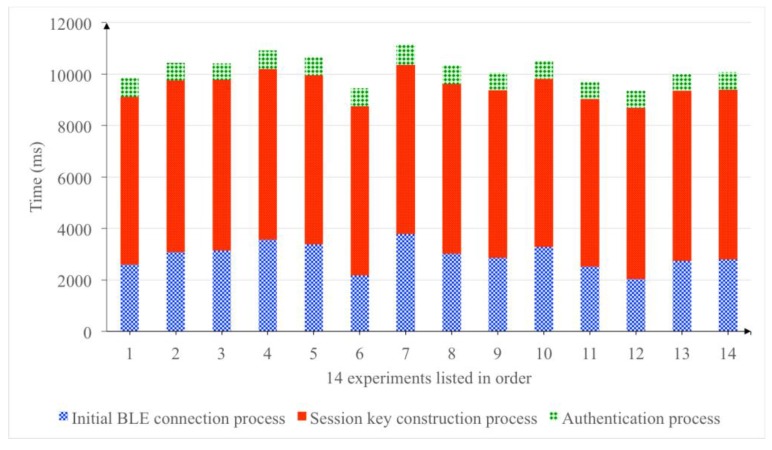
The time consumption of the prototype to establish one payment session in 14 different experiments.

When the prototype is executed to establish a session, almost 64% of the time is consumed during the session key construction phase. Meanwhile, the initial BLE connection process such as the BLE service discovery and pairing process consumes 29% of the total processing time, and the remaining 7% of the time is used for the authentication phase. Figure 14 shows the time consumption of the prototype to establish one payment session in fourteen experiments.

**Figure 15:**
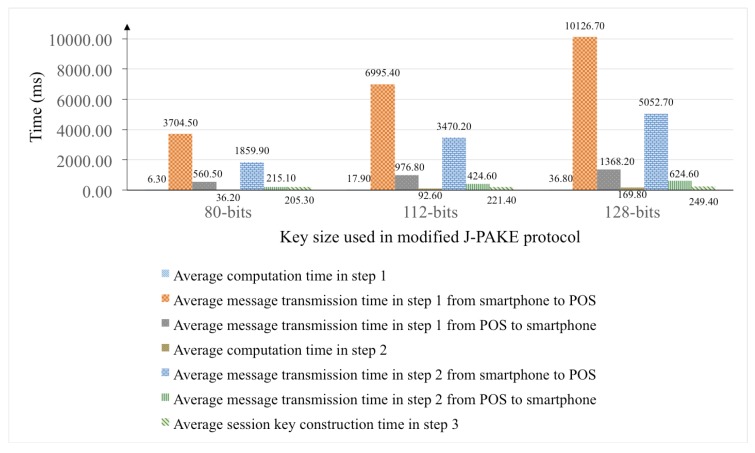
The average duration of each step in session key construction phase over three different key sizes in modified J-PAKE protocol.

Within the same set of experiments, the construction process of 80-bits session key takes around six to seven seconds on average. In order to observe the behavior of modified J-PAKE protocol used in the session key construction phase, further experiments are conducted by observing the time consumption for each step in the session key construction phase. Figure 15 shows the average duration of each step in the session key construction phase based on the key size used in the modified J-PAKE protocol. In these experiments, three key sizes are chosen: 80-bits, 112-bits, and 128-bits.

With the key size set to 80-bits in Figure 15, it shows that the messages transmission time between the user’s smartphone and the POS terminal during the session key construction phase consumes 6340 ms. Nevertheless, the total time consumption for the session key construction phase is 6587.8 ms. It indicates that 96% of the total time consumption is spent on the message transmission through Bluetooth channel. Based on the BLE core specification 4.2 [7], the theoretical bandwidth of BLE 4.0 and BLE 4.1 is up to 236.7 kbps. However, the measured bandwidth of BLE 4.0 and BLE 4.1 using a CC2540 Bluetooth sniffer [39] is only 50 kbps in our experiments. In comparison with BLE 4.0 and BLE 4.1, new BLE 5.0 specification [40] has increased the bandwidth capacity from 236.7 to 1400 kbps, which is 5.91 times larger. Even though there is no device equipped with BLE 5.0 module currently, it is assumed that the practical bandwidth of BLE 5.0 will be around 5.91 times larger than the previous BLE 4.0 and BLE 4.1. If the transmission bandwidth of BLE module can be raised up to 5.91 times larger, then the time consumption for message transmission in the session key construction phase can be reduced to 1072.75 ms.

**Figure 16:**
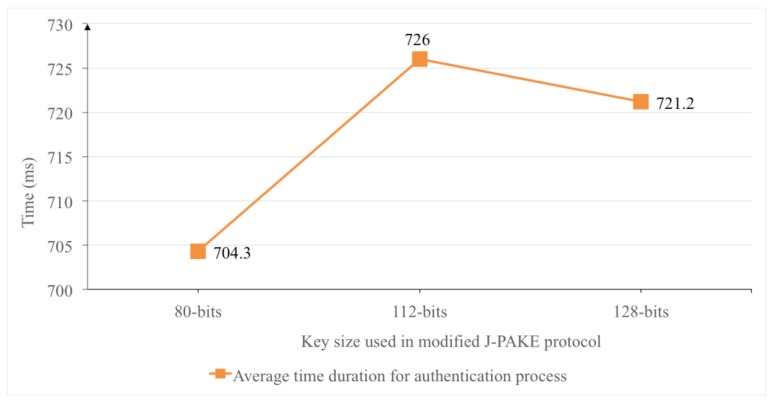
The average time duration of the authentication process over three different key sizes in the modified J-PAKE protocol.

Based on this assumption, the total time consumption for the session key construction phase can be reduced to 1320.55 ms. The average time for authentication process with the key size set to 80-bits is 704.3 ms as shown in Figure 16. In terms of the time consumption for initial connection of two BLE modules in our experiments, it is around 3 s in average as shown in Figure 14. Nevertheless, the time required to establish initial connection between two BLE modules is inevitable and hard to reduce without introducing new BLE hardware module. In brief, with new BLE 5.0 module, the proposed user authentication mechanism could establish a secure payment session with dynamically constructed session key around 2 s without counting the time consumption of BLE channel establishment. 

On the other hand, the increase of key size in the modified J-PAKE protocol does not have much effect on time consumption of the authentication phase in the proposed system. The time consumption between 80-bits, 112-bits and 128-bits of key sizes in the modified J-PAKE protocol only produces a slight 5–22 ms difference on the duration of the authentication process. Figure 16 shows the average time duration of authentication process over different key sizes used in the modified J-PAKE protocol.

## 6. Security Analysis of the Proposed Authentication Protocol

Security analysis for the proposed authentication protocol is conducted to evaluate mutual authentication and the security strength of session key generation. In addition, the proposed protocol defends against impersonation attack, replay attack, and man-in-the-middle attack. We assume the cryptographic hash function used in the proposed protocol is able to withstand all known types of cryptanalytic attacks. In other words, the hash function has the characteristics of collision resistance, and is able to counteract the pre-image attacks and the second pre-image attacks. Moreover, a Cryptographically Secure Pseudorandom Value Generator (CSPRVG) is assumed to be used to generate all the random values in the proposed protocol. The CSPRVG satisfies all statistical tests and the output value is unpredictable to attackers.

**Theorem** **1.** The proposed protocol supports mutual authentication.

**Proof.**  Due to the computational difficulty of the Diffie-Hellman problem, the secret value to construct session secret Q can be computed only by legitimate customer’s smartphone and legitimate POS terminal. Both the legitimate customer’s smartphone and legitimate POS terminal will use the following formula $Q={\left(\beta {\left(X_{4}\right)}^{-x_{2}K_{a}}\right)}^{x_{2}}$ and $Q={\left(\alpha {\left(X_{2}\right)}^{-x_{4}K_{a}}\right)}^{x_{4}}$ respectively; then both of them use the session secret Q to create a unique session key $K_{s}$ and a unique nonce $N_{s}$ at both ends. Next, the legitimate customer’s smartphone could compute hash key $U_{1}=K_{s}||r_{1}$ and the hashed message $P_{1}=H_{U_{1}}\left(\left(r_{1}⊕\left(K_{s}||n\right)\right)||ID_{c}\right)$. The hashed message $P_{1}$ is sent to the legitimate POS terminal afterwards. After receiving $P_{1}$, the legitimate POS terminal computes the random value $r_{1}{}^{'}=\left(r_{1}⊕\left(K_{s}||n\right)\right)⊕\left(K_{s}||n\right)$ and the hash key $U_{2}=K_{s}||r_{1}{}^{'}$. The random value $r_{1}{}^{'}$ and the hash key $U_{2}$ are used by the POS terminal to authenticate the customer’s smartphone by evaluating whether the received message value $P_{1}$ is equivalent to the hashing computation result $H_{U_{2}}\left(\left(r_{1}{}^{'}⊕\left(K_{s}||n\right)\right)||ID_{c}\right)$.

For the legitimate customer’s smartphone to authenticate the POS terminal, the POS terminal first computes a secret value $W_{2}=\left(r_{2}⊕r_{1}{}^{'}\right)⊕\left(K_{s}||n\right)$ and a hash key $U_{3}=K_{s}||r_{2}$. The secret value $W_{2}$ and the hash key $U_{3}$ are used to produce a hashed message $P_{2}=H_{U_{3}}\left(r_{1}{}^{'}\left|\left|r_{2}\right|\right|W_{2}||ID_{p}\right)$, in which the hashed message $P_{2}$ will be sent to the legitimate customer’s smartphone. After receiving $W_{2}$ and $P_{2}$, the legitimate customer’s smartphone could compute the random value $r_{2}{}^{'}=\left(W_{2}⊕\left(K_{s}||n\right)\right)⊕r_{1}$ and the hash key $U_{4}=K_{s}||r_{2}{}^{'}$. Next, the legitimate customer’s smartphone authenticates the POS terminal by evaluating whether the received message value $P_{2}$ is equivalent to the hashing computation result $H_{U_{4}}\left(r_{1}||r_{2}{}^{'}\left|\left|W_{2}\right|\right|ID_{p}\right)$. Based on these two proofs, it can be concluded that the proposed protocol supports mutual authentication. ☐

**Theorem** **2.** The proposed protocol provides session key security.

**Proof.**  Assume that an adversary 𝒜 learns a session key $K_{s}{}^{prev}$ from the previous successful session. The adversary 𝒜 cannot derive the previous secret value $Q^{prev}$ from the session key $K_{s}{}^{prev}$ because the output of random string extension function $R_{ext}$ cannot be used to easily derived its input parameter $Q^{prev}$. The only way to derive the value of $Q^{prev}$ is to apply brute-force computation to find the matched value of $Q^{prev}$ based on the session key construction formula $K_{s}{}^{prev}=R_{ext}\left(salt,Q^{prev}\right)$. Even if the adversary 𝒜 could successfully derives the $Q^{prev}$, it is impossible for the adversary to generate the current secret value Q from the previous secret value $Q^{prev}$ as the value of Q is constructed from the values of $y_{1},\;x_{1},\;x_{2},x_{3}$ and $x_{4}$, which are always freshly generated for each session.

As $K_{s}=R_{ext}\left(salt,\;Q\right)$ and $Q={\left(\beta {\left(X_{4}\right)}^{-x_{2}K_{a}}\right)}^{x_{2}}={\left(\alpha {\left(X_{2}\right)}^{-x_{4}K_{a}}\right)}^{x_{4}}$, the other way for the adversary to derive the current session key $K_{s}$ is by directly computing the values of Q, $X_{1}=g^{x_{1}}$, $X_{2}=g^{x_{2}}$, $X_{3}=g^{x_{3}}$, $X_{4}=g^{x_{4}}$ and $K_{a}=H\left(SSC\left(A_{i}^{p}\right)||\left(y_{1}||ID_{c}\right)\right)$. As both the customer’s smartphone and the POS terminal always generate new random values $y_{1},\;x_{1},\;x_{2},x_{3}$ and $x_{4}$ for each session and Q is generated based on key agreement mechanism derived from Diffie-Hellman problem, hence, the adversary 𝒜 needs to solve the Diffie-Hellman problem to acquire the current Q. ☐

**Theorem** **3.** The proposed protocol defends against impersonation attack.

**Proof.**  Assume that an adversary 𝒜 impersonates the client (the customer’s smartphone) to establish a secure session with the legitimate POS terminal. The adversary 𝒜 must have the symmetric key of a legitimate customer $K_{c}$, which is obtained by the legitimate customer during the registration phase of the system. The symmetric key $K_{c}$ is stored in the secure element of the smartphone; therefore, it is impossible for the adversary 𝒜 to learn the correct symmetric key $K_{c}$ from a legitimate customer’s smartphone. Hence, the adversary cannot decrypt the encrypted value $V_{2}=E_{K_{c}}\left(K_{a}||MAC_{p}\right)$ sent by the TTP server during the initialization phase. Therefore, the adversary 𝒜 is unable to initiate a genuine secure session with the legitimate POS terminal. The BLE connection initiated by the adversary 𝒜 to the legitimate POS terminal will always be refused, because the adversary 𝒜 cannot derive the user secret $K_{a}$ constructed by the TTP server. The adversary 𝒜 is not able to construct a genuine session key $K_{s}=R_{ext}\left(salt,\;Q\right)$, because the session secret Q is constructed using the user secret $K_{a}$ as one of its input parameters in this formula $Q={\left(\beta \cdot X_{4}{}^{-x_{2}\cdot K_{a}}\right)}^{x_{2}}={\left(\alpha \cdot X_{2}{}^{-x_{4}\cdot K_{a}}\right)}^{x_{4}}$.

The other case is that where an adversary 𝒜 launches an impersonation attack to make itself appear as a POS terminal to legitimate customer’s smartphones. Then, the adversary 𝒜 needs to have the symmetric key $K_{p}$ of a legitimate POS terminal to decrypt the encrypted message $V_{3}=E_{K_{p}}\left(K_{a}||ID_{c}\right)$ and obtain the correct user secret $K_{a}$ sent by the TTP server during the initialization phase. As the adversary 𝒜 could not learn the correct user secret $K_{a}$, then the adversary 𝒜 would be unable to construct the proper session secret $Q={\left(\beta \cdot X_{4}{}^{-x_{2}\cdot K_{a}}\right)}^{x_{2}}={\left(\alpha \cdot X_{2}{}^{-x_{4}\cdot K_{a}}\right)}^{x_{4}}$. Once the adversary 𝒜 is unable to construct the correct session secret Q, he would be unable to generate the session key $K_{s}$ from the random string extension function $R_{ext}\left(salt,\;Q\right)$. ☐

**Theorem** **4.** The proposed protocol defends against replay attack.

**Proof.**  Assume an adversary 𝒜 has successfully learnt the previous user secret $K_{a}^{prev}$ to perform a replay attack. The adversary 𝒜 will send two message sets $M_{1}$ and $M_{3}$ to the legitimate POS terminal during the session key construction phase, in which the message set $M_{3}$ is generated with the previous user secret $K_{a}^{prev}$. The two message sets are used to construct a session secret Q for generating the current session key $K_{s}$. However, the legitimate POS terminal will receive a different value of user secret $K_{a}^{curr}$ from the TTP server since a new random value $y_{1}$ is used to construct user secret $K_{a}^{curr}=H\left(SSC\left(A_{i}^{p}\right)\left|\left|\left(y_{1}||ID_{c}\right)\right|\right|TS\right)$ for the current session. Hence, the session secret $Q={\left(\beta \cdot X_{4}{}^{-x_{2}\cdot K_{a}^{prev}}\right)}^{x_{2}}$ generated by the adversary 𝒜 is not equivalent to the session secret $Q={\left(\alpha \cdot X_{2}{}^{-x_{4}\cdot K_{a}^{curr}}\right)}^{x_{4}}$ generated by the legitimate POS terminal. Therefore, the session key $K_{s}^{adv}$ generated by the adversary will not be equivalent to the session key $K_{s}^{POS}$ generated by the legitimate POS terminal. In consequence, the replay attack cannot launch successfully.

In case the adversary 𝒜 performs a replay attack during the authentication phase of the payment process, the adversary 𝒜 should have the encrypted message $W_{1}=r_{1}⊕\left(K_{s}^{prev}||n\right)$, and the hashed value $P_{1}=H_{U_{1}}\left(W_{1}||ID_{c}\right)$ from the previous session. When the adversary 𝒜 performs the replay attack, the POS terminal will know that the adversary 𝒜 does not use the current session key $K_{s}^{curr}$ because the received hashed value $P_{1}$ is not equivalent to the calculated hashed value $H_{U_{2}}\left(W_{1}||ID_{c}\right)$, where the hash key $U_{2}=\left(K_{s}^{curr}||r_{1}\right)$. As the result, the replay attack cannot launch successfully. Even if the adversary 𝒜 performs the replay attack using the encrypted message $W_{3}=E_{U_{5}}\left(M,\;TS\right)$ from the previous session, the POS terminal is able to detect the timestamp TS used in the received encrypted message $W_{3}$ has already expired. ☐

**Theorem** **5.** The proposed protocol defends against man-in-the-middle attack.

**Proof.**  Based on the proof given from Theorem 1, the proposed protocol provides mutual authentication between the legitimate user’s smartphone and the legitimate POS terminal. Hence, the proposed authentication protocol is able to defend against man-in-the-middle attack. □

## 7. Conclusions

New mobile payment systems are emerging in recent years such as NFC-based payment systems and smart card based payment systems (i.e., the card is embedded in a smartphone). As NFC-embedded smartphones are more expensive than ordinary ones in general, proposing a novel mobile payment system for most people who only have low-end or medium-end smartphones is a practical user demand. Since most smartphones have embedded BLE modules to support communication with nearby devices through Bluetooth technology, it is plausible to construct a BLE-based mobile payment system for common people. In this paper, we introduced a BLE-based mobile payment system such that people carrying BLE-enabled wearable devices can perform Over-the-Air payment actions through a Bluetooth communication channel between their wearable devices and the BLE-enabled POS terminal. To secure the payment process, an indoor-positioning based authentication protocol is proposed to dynamically generate the session key for each user payment session, in which the session key is used by both communicating parties to encrypt and decrypt payment information transmitted between them. The session key is generated from the user secret, which is created by the Trusted Third Party server with a set of received Bluetooth beacon signals indicating the current position of the user. The proposed protocol includes three phases: initialization phase, session key construction phase, and authentication phase. Two indoor positioning algorithms, a trilateration algorithm and a least squares algorithm, are adopted to determine whether the user is at the payment-enabled area.

Based on the proposed authentication protocol, a prototype of the mobile payment system is implemented and the system performance is evaluated. According to the experiment results, the indoor positioning algorithms shows an acceptable accuracy rate for determining whether the customer is located within the payment-enabled area. The best accuracy rate for customer position prediction is 97.29% when using the least squares algorithm combined with an average filter. As the bandwidth of BLE 4.0 and 4.1 modules is only up to 236.7 kbps, the time consumption for message transmission during user authentication is heavy. With the session key set at 80 bits, the total communication cost is 6.5340 s on average. Therefore, the prototype requires 6.5878 s to accomplish one session authentication on average. By applying a new BLE 5.0 module with 1400 kbps bandwidth, we estimate the proposed system should be able to accomplish a mutual authentication operation within 2 s.

Security analysis is conducted to evaluate the security strength of the proposed protocol. Based on the analysis results, the customer’s smartphone and the communicating POS terminal can securely and mutually authenticate each other. In addition, an adversary cannot derive or successfully guess the session key, which will be generated for the next authentication session between two communicating parties, based on the knowledge learned from the previous session key. Moreover, the proposed protocol withstands impersonation attacks, replay attacks, and man-in-the-middle attacks.

## Figures and Tables

**Table 1:** Notations used in the proposed authentication protocol.

Notation	Definition
$ID_{c},\;ID_{p},ID_{TTP}$	The identities of the customer’s wearable device ($ID_{c}$), the POS terminal ($ID_{p}$), and the Trusted Third Party server $\left(ID_{TTP}\right)$.
$A_{i}^{p}$	A set contains of the unique identifier of beacon tag i and the corresponding partial secret of POS terminal p stored in the beacon tag i, where 1≤i≤4.
$MAC_{p}$	The MAC address of POS terminal p.
M	A message that contains the payment transaction information.
TS	The value of current timestamp generated from the customer’s wearable device.
$K_{p},\;K_{c}$	The symmetric keys of the POS terminal p and the customer’s wearable device c, correspondingly.
$r_{1},r_{2},x_{1},x_{2},x_{3},x_{4},y_{1}$	Random values generated by Random Value Generator (RVG).
$K_{a}$	The user secret generates by the TTP server for the current authentication session based on the position of customer’s wearable device and the received four partial POS terminal secrets.
$K_{s}$	A session key dynamically and individually generates by the customer’s wearable device and the POS terminal during the session key construction phase.
n	A nonce generates from the $R_{ext}$ function in the session key construction phase.
SSC()	A Shamir secret sharing construction function.
PK()	A Zero Knowledge Proof function as defined in [13].
VK()	A verification function for the Zero Knowledge Proof as defined in [13].
$R_{ext}()$	A random string extension function, in the proposed protocol the fixed string output length of this function is 256 bytes.
H()	A one-way hash function.
$H_{k}()$	A one-way keyed hash function with key k.
$E_{k}()$	An encryption function using key k.
$D_{k}()$	A decryption function using key k.

**Table 2:** Devices used to build the prototype.

Device Name	Hardware Specifications
Samsung Galaxy S5	Quad core 2.5 GHz 2 GB RAM Android OS v6.0.1 (Marshmallow) BLE 4.0
Samsung Galaxy S6	Octa core 4 × 2.1 GHz and 4 × 1.5 GHz 3 GB RAM Android OS v6.0.1 (Marshmallow) BLE 4.1
Estimote proximity beacon tag	64 MHz ARM 512 kB flash and 64 kB RAM Built-in 2.4 GHz BLE Support iBeacon and Eddystone frame format

**Table 3:** A total of 148 experiments for indoor positioning detection using least squares algorithm.

**(a) The Experimental Results without Using Any Filter**	
Number of true predictions	69
Number of false predictions	5
Total number of experiments	74
Accuracy rate	93.24%
**(b) The Experimental Results Using an Average Filter**	
Number of true predictions	72
Number of false predictions	2
Total number of experiments	74
Accuracy rate	97.29%

**Table 4:** A total of 148 experiments for indoor positioning detection using trilateration algorithm.

**(a) The Experimental Results without Using Any Filter**	
Number of true predictions	58
Number of false predictions	16
Total number of experiments	74
Accuracy rate	78.37%
**(b) The Experimental Results Using an Average Filter**	
Number of true predictions	54
Number of false predictions	20
Total number of experiments	74
Accuracy rate	72.97%
